# Impact of Tympanostomy Tube Placement on Pediatric Developmental Outcomes: A Systematic Review

**DOI:** 10.7759/cureus.98711

**Published:** 2025-12-08

**Authors:** Alexander N Fahmy, Doreen Lam, Melanie Cedrone, Kavita Dedhia

**Affiliations:** 1 Department of Otolaryngology, Drexel University College of Medicine, Philadelphia, USA; 2 Department of Otolaryngology, Perelman School of Medicine, Philadelphia, USA; 3 Holman Biotech Commons, University of Pennsylvania Health System, Philadelphia, USA; 4 Department of Otorhinolaryngology, University of Pennsylvania Health System, Philadelphia, USA; 5 Department of Otolaryngology, Children's Hospital of Philadelphia, Philadelphia, USA

**Keywords:** developmental measurement instruments, developmental outcomes, otitis media, pediatrics, tympanostomy tubes

## Abstract

The goal of this study was to systematically review the literature on developmental outcomes following tympanostomy tube insertion (TTI) in pediatric patients with otitis media (OM) and evaluate whether TTI improves language, cognitive, or behavioral functioning compared to conservative management. A systematic review was conducted to identify studies reporting developmental outcomes after TTI in children with OM. Two independent reviewers screened titles, abstracts, and full-text articles using predefined inclusion criteria. Risk of bias was assessed with the Cochrane Risk of Bias 2 tool and MINORS criteria. Of 2,746 studies identified, 13 met inclusion criteria. Study designs included three randomized controlled trials (RCTs), nine prospective cohort studies, and one retrospective study. Ten different developmental measurement instruments (DMIs) were used, targeting cognitive, language, and behavioral domains. Only two RCTs demonstrated statistically significant short-term improvements in developmental outcomes after TTI, particularly in personal-social skills and behavior at nine months post-intervention. However, these gains were not sustained at 18 months. One observational study reported worsening behavioral outcomes after TTI. The remaining studies, including one RCT and several large cohorts, showed no developmental benefit. Heterogeneity in DMIs, inconsistent follow-up duration, and limited demographic and socioeconomic data impeded comparison and synthesis. Evidence supporting developmental improvement following TTI in pediatric OM patients is limited and mixed. While short-term gains are noted in select RCTs, these findings are not consistently replicated. Future research should prioritize standardized outcome measures, better demographic reporting, and clearer stratification by developmental risk to clarify TTI's impact on child development.

## Introduction and background

Otitis media (OM) is one of the most common reasons for pediatric healthcare visits in the United States. It encompasses both acute otitis media (AOM) and otitis media with effusion (OME). AOM has a peak incidence between six months and two years of age; by age 3, approximately 80-90% of children will have experienced at least one episode [1]. OME, characterized by the presence of middle ear effusion without acute symptoms, will be experienced at least once by approximately 80% of children by four years of age [2].

OM management ranges from medical management (MM) and watchful waiting (WW) to tympanostomy tube insertion (TTI). TTI is one of the most common surgical procedures in children worldwide and is an option for children with recurrent AOM or persistent OME. The 2022 AAO-HNS guidelines emphasize prompt evaluation for TTI in children who are considered at risk for speech, language, or developmental delays, highlighting the importance of developmental vulnerability in surgical decision-making [3].

Children with OME may experience temporary hearing loss, which has been associated with behavioral issues and developmental concerns. A growing body of evidence suggests that early and prolonged OME during critical periods of development may negatively influence language acquisition and academic performance. For example, a birth cohort study by Bennett et al. [4] linked OME to increased inattention, hyperactivity, lower IQ, and reduced reading scores. Still, it remains unclear whether surgical intervention with TTI can reverse or prevent these outcomes.

Several studies have attempted to evaluate developmental outcomes following OM and TTI using tools such as cognitive assessments, language testing, behavioral inventories, and academic benchmarks. Yet, findings remain inconsistent, and there is no consensus on whether TTI confers a developmental advantage over WW or MM.

Given this heterogeneity, definitive conclusions about the developmental impact of TTI remain elusive. TTI is effective for reducing OME-related conductive hearing loss and decreasing infection frequency, both of which can influence developmental pathways, yet the magnitude and durability of such effects differ across children. Better-quality evidence is needed to clarify these relationships.

This systematic review aims to synthesize the existing literature on TTI and its impact on developmental outcomes in children, with a focus on comparing early surgical intervention to conservative management strategies in populations at risk for developmental delays.

## Review

Methods

To evaluate the effect of TTI on developmental outcomes, we performed a systematic review of literature on pediatric patients (<18 years old) with OM and TTI, with outcomes measured using validated developmental tests or developmental measurement instruments (DMIs). Ten different measurement tools were used, though only two were employed across multiple studies. These tools varied in the specific outcomes they measured, but all focused on assessing reading performance and IQ. This systematic review was reported in accordance with the Preferred Reporting Items for Systematic Reviews and Meta-Analyses (PRISMA) 2020 statement.

Literature search strategy

A literature search of PubMed, Embase (Elsevier), Scopus (Elsevier), and Cochrane Library (Wiley) was performed for articles published from inception to February 12, 2024. The search was then updated on April 14, 2025. An information specialist (M.C.) was consulted to develop the search strategy, which involved a combination of controlled vocabulary and keywords ('otitis media', 'middle ear inflammation', 'middle ear infection', 'serous otitis', 'quality of life', 'health-related quality of life', 'developmental outcomes', 'tympanostomy tubes', 'middle ear ventilation', 'grommet', 'prospective studies', 'randomized controlled trial'). The search was limited to articles published in English. Covidence was used for deduplication and screening.

Selection criteria

The inclusion criteria for this review included studies of children aged 0 to 18 years with AOM, recurrent AOM (rAOM), OME, or middle ear effusion (MEE) who had undergone TTI and reported developmental outcomes. Exclusion criteria included non-English language studies, studies including adults, non-validated DMIs, and studies with patients undergoing TTI with adenoidectomy or adenotonsillectomy. Two investigators (D.L. and A.F.) independently reviewed study abstracts, and a subsequent review of full-length articles was performed to meet the above eligibility criteria. Any discrepancies encountered during each stage of the dual independent investigator review were resolved through discussion.

Data extraction

Descriptive characteristics were extracted from each study, including the country of origin, study design, sample size, age range, sex, race, parental income and education levels, comorbidities, otitis media (OM) subtype, surgical intervention, follow-up duration, and developmental outcomes. Analysis of developmental outcomes was complicated by the heterogeneity of assessment tools used across studies. Consequently, each study was individually analyzed based on the specific survey or tool employed. The results were then reviewed to determine whether the study reported an overall improvement in developmental outcomes.

Quality assessment

Randomized controlled trials (RCTs) were assessed for internal validity using the Cochrane Risk of Bias 2 tool, while prospective and retrospective studies were evaluated using the Methodological Index for Non-Randomized Studies (MINORS) criteria.

Results

Literature Search and Risk of Bias Assessment

**Figure 1:**
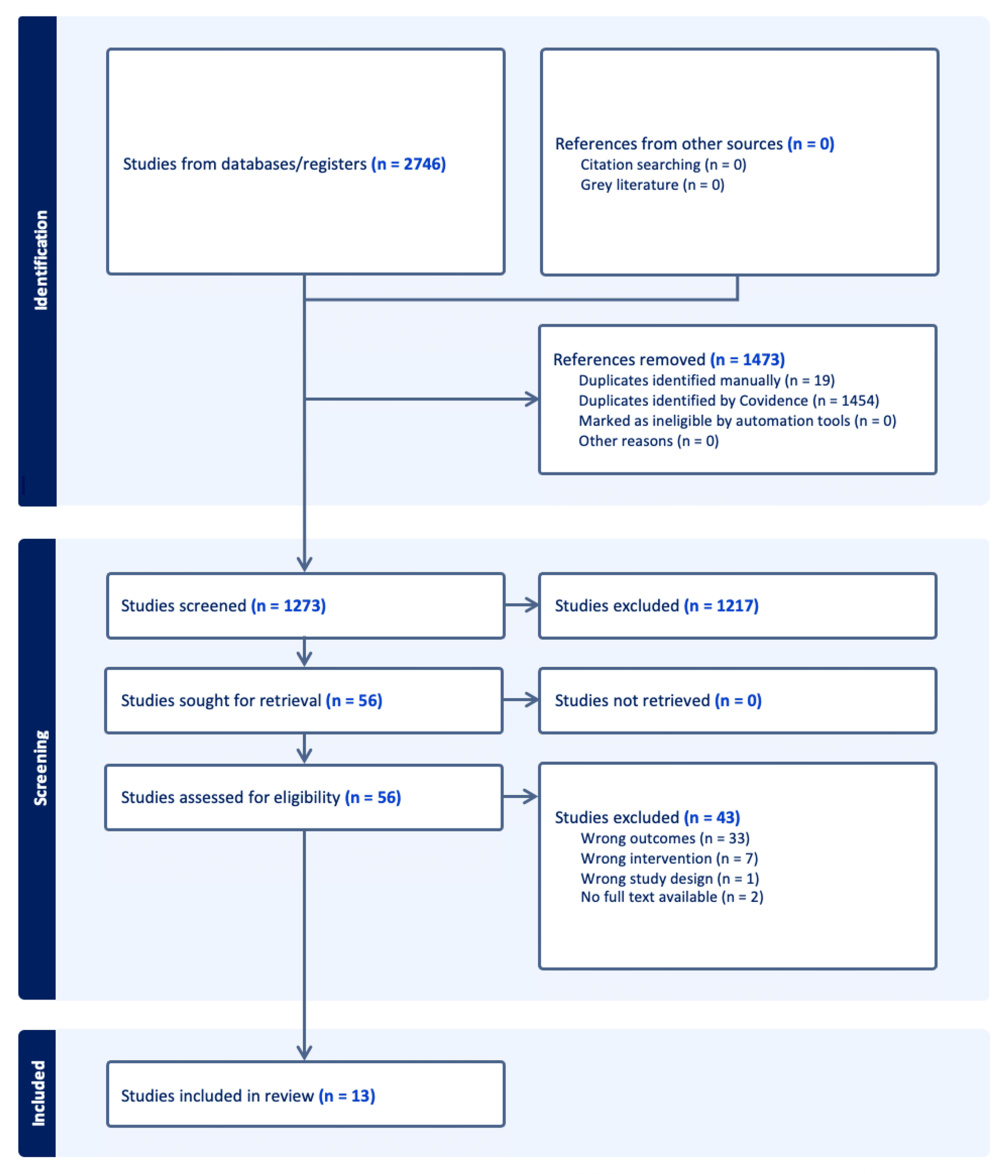
PRISMA study diagram. PRISMA: Preferred Reporting Items for Systematic Reviews and Meta-Analyses.

The initial database search returned 2,746 studies with 1,473 duplicates, leaving 1,273 for review. A total of 1,217 were excluded after title and abstract review, and the remaining 56 studies underwent full-text review. Of these, 43 studies were excluded for reasons such as lack of relevant developmental outcomes (n=33), incorrect study design (n=1), alternative interventions (n=7), and unavailability of full article text (n=2). Thirteen studies met the criteria for inclusion (Figure 1). 

**Figure 2:**
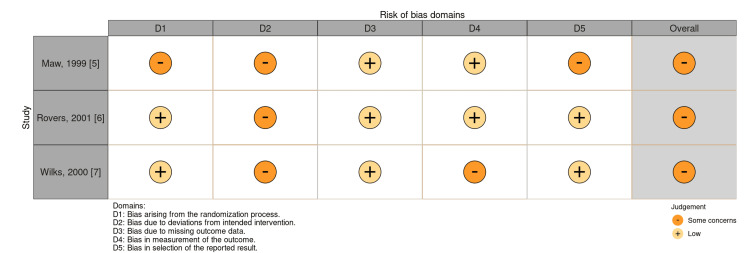
ROBVIS tool. Used only for the three RCTs. ROBVIS: Risk-Of-Bias VISualization; RCTs: randomized control trials.

The risk of bias assessment for each of the three RCTs [5-7] indicated some concerns, with all studies showing issues in domain 2, related to deviations from the intended interventions. Of the RCTs, the study by Maw et al. [5] also showed concerns in domains 1 and 5, randomization and bias with the reported result, respectively. The study by Wilks et al. [7] also showed concerns in domain 4, bias in the measurement of the reported outcome (Figure 2). 

MINORS criteria were used to assess the methodological quality of non-randomized studies [8]. Most studies were of moderate quality, with the main concerns being the lack of unbiased assessment of the study endpoint and loss to follow-up <5% (Table 1).

**Table 1:** MINORS criteria. MINORS: Methodological Index for Non-Randomized Studies.

Author, Year	1. A clearly stated aim	2. Inclusion of consecutive patients	3. Prospective collection of data	4. End points appropriate to the aim of the study	5. Unbiased assessment of the study endpoint	6. Follow-up period appropriate to the aim of the study	7. Loss to follow-up <5%	8. Prospective calculation of the study size	Total
Paradise et al., 2003 [9]	2	2	2	2	0	2	1	2	13
Paradise et al., 2001 [10]	2	2	2	2	0	2	1	2	13
Paradise et al., 2007 [11]	2	2	2	2	0	2	1	2	13
Paradise et al., 2005 [12]	2	2	2	2	0	2	1	2	13
Niclasen et al., 2016 [13]	2	0	2	2	0	0	1	2	9
Herzog et al., 2020 [14]	2	2	2	2	0	0	2	2	12
Peters et al., 1994 [15]	2	2	2	2	2	2	1	2	15
Schilder et al., 1997 [16]	1	1	2	2	0	2	0	1	9
Barry et al., 2019 [17]	2	2	2	2	0	0	0	2	10
Rach et al., 1991 [18]	2	0	2	2	0	2	0	0	8

Study Demographics & Characteristics

Eight studies were conducted in English-speaking countries. Most studies were prospective cohort (9), three RCTs, and one was a retrospective cohort study. The median follow-up for the RCTs was 18 months (range 3 months to 11 years). The non-RCTs were generally vague in reporting follow-up durations (Table 2).

**Table 2:** Study design. TTI: tympanostomy tube insertion; OME: otitis media with effusion; RCT: randomized controlled trial.

Author (Year)	Study design	Intervention (n)	Timing of intervention	Comparison group	Outcome measures	Follow-up duration
Barry et al., 2019 [17]	Prospective cohort	TTI (25)	—	Active observation (20), Normal hearing (45)	OMQ-14, ECLIPS	3 months
Rach et al., 1991 [18]	Prospective cohort	TTI (22)	—	OME without treatment (21), No OME (9)	Reynell Development Language Scales	24 months
Niclasen et al., 2016 [13]	Retrospective cohort	TTI (999)	—	No OME (3585), 4+ OME without TT (569), 1–3 OME without TT (2425)	SDQ (Danish)	10-12 years
Maw et al., 1999 [5]	RCT	TTI (92)	Within 6 weeks AD	Watchful waiting (90)	PSI/SF, McCarthy Scales, Peabody, NDW, MLUm, PCC-R, CBCL	3 years
Paradise et al., 2007 [11]	Prospective cohort	TTI (429)	Immediate (n=164), Delayed (n=88)	Non-randomized subgroups	Woodcock Reading Mastery, Woodcock–Johnson III	9–11 years
Paradise et al., 2001 [10]	Prospective cohort	TTI (429)	Immediate (n=216), Delayed (n=213)	—	PSI/SF, McCarthy Scales, Peabody, NDW, MLUm, PCC-R, CBCL	3 years
Paradise et al., 2003 [9]	Prospective cohort	TTI (407)	Immediate, Delayed	—	Same as above	3 years
Paradise et al., 2005 [12]	Prospective cohort	TTI (395)	Immediate (170), Delayed (81)	Non-randomized subgroups	Wechsler, SCAN, Nonword repetition, PSI/SF, Peabody, NDW, MLUm, PCC-R, CBCL	6 years
Rovers, 2001 [6]	RCT	TTI (93)	—	Watchful waiting (94)	TaiQol, Erikson Scales	12 months
Wilks et al., 2000 [7]	RCT	TTI (92)	Within 6 weeks AD	Watchful waiting (90)	Richman Behavior Checklist	18 months
Schilder et al., 1997 [16]	Prospective cohort	TTI (56)	3 months AD	No surgery (102)	Language and cognitive tests	7–8 years
Peters et al., 1994 [15]	Prospective cohort	TTI (37)	—	OME without surgery (151), No OME (82)	Literacy and cognitive tests	3–5 years
Herzog et al., 2020 [14]	Prospective cohort	TTI (12,826)	—	No surgery (19,233)	SDQ (Danish)	7 years

The study size ranged from 52 to 52,877 patients. Of note, four Paradise studies [8-11] shared the same study population, as the investigators followed a birth cohort over time with evaluations at predetermined intervals. Sociodemographic information was infrequently reported, with three studies reporting race/ethnicity, three reporting education levels, and two reporting insurance type (Table 3). 

**Table 3:** Study demographics. MEE: middle ear effusion; OME: otitis media with effusion.

Author, Year	Country	Sample size	Condition	Age (range/mean)	Sex (M/F)	Race/ethnicity	SES/education	Insurance
Barry et al., 2019 [17]	UK	96	OME	2–12 years (mean 5.7)	56/34	—	—	—
Rach et al., 1991 [18]	Netherlands	52	OME	—	—	—		Medicaid (n=518), Private (n=280)
Niclasen et al., 2016 [13]	Denmark	—	OME	11 months–15.4 years (mean NR)	3854/3694	—	—	—
Maw et al., 1999 [5]	UK	186	OME	3.7 years and 4.6 years	XX/94	—	—	—
Paradise et al., 2001 [10]	USA	6350	MEE	—	229/173	—		Medicaid (n=400), Private (n=321)
Paradise et al., 2003 [9]	USA	6350	MEE	Tested at 4 years	345/286	White (453), Black (182), Other (14)	Attended college (74.6%)	—
Paradise et al., 2005 [12]	USA	6350	MEE	Tested at 3, 4, and 6 years	397/332	White (478), Black (243), Other (18)	—	—
Paradise et al., 2007 [11]	USA	6350	MEE	Mean 9.5 years	299/219	White (296), Black (207), Other (15)	—	—
Rovers et al., 2001 [6]	Netherlands	1081	OME	1–2 years (mean 1.6)	—	—	—	—
Wilks et al., 2000 [7]	UK	182	OME	Preschool-aged (mean NR)	—	—	—	—
Schilder et al., 1997 [16]	Netherlands	1328	OME	2–4 years	15/12	—	Maternal education levels: 5 (lowest) to 1 (highest)	Yes (n=273,555), No (n=11,801)
Herzog et al., 2020 [14]	Denmark	52,877	OM	Followed for 7 years	26/25,991	—	—	—

Development Measurement Instruments

Ten different DMIs were used across the 13 included studies. Various developmental outcomes were tested, including language, cognition, emotional and behavioral skills, and motor skills. Overall, the length of the DMIs varied significantly, with assessments containing between 38 and 100 items. The time required to complete these assessments varied based on the assessment administered and age group of the child. There was limited consistency among studies in the developmental DMIs that were chosen, with only three being used in multiple studies. Three studies [5,9,10] used the McCarthy Scales of Children’s Abilities, which assess verbal, perceptual performance, quantitative, general cognitive, memory, and motor skills. Two studies [12,13] used the Danish SDQ, which monitors emotional difficulties, conduct problems, hyperactivity/inattention, peer problems, and prosocial behavior, reporting worsening behavioral and learning difficulties later in childhood. Two studies [14,15] focused on specific ability tests: one measured verbal language comprehension, assessing a child's understanding of spoken language, while the other focused on spelling and reading, evaluating literacy development and its relationship to overall academic success. DMIs that were used in only one study included the Wechsler Intelligence Scale for Children (WISC), the Woodcock-Johnson III Tests of Achievement, the Erikson Scales, the Reynell Developmental Language Scales, the ECLIPS scales, and the Richman Behavior Checklist (Table 4). 

Developmental Outcomes After TTI

Developmental outcomes improved after TTI in two RCTs [5,7]. The study by Maw et al. [5] showed a significant improvement in personal-social skills at nine months in the TTI group compared to the watchful waiting group (p = 0.04), based on standardized assessment using the Griffiths scale. Verbal comprehension and expressive language scores also favored TTI at nine months with statistically significant improvements (p=0.045), but this difference was no longer statistically significant at 18 months. Other developmental domains, including locomotor function, hearing and speech, eye-hand coordination, and practical reasoning, did not differ significantly between groups. Similarly, the study by Wilks et al. [7] reported early behavioral improvements at nine months post-intervention using the Richman Behavior Checklist, which considers a score >10 as indicative of behavioral concerns. Scores in the TTI and control groups equalized at the 18-month assessment. Both studies relied on structured, validated tools completed by researchers or clinicians, reducing subjective bias and allowing for more consistent comparisons.

No significant postoperative improvement was identified in 10 other studies, one RCT and nine prospective cohort studies. Niclasen et al. [13] reported worsening in behavioral outcomes following TTI, particularly among children with four or more episodes of otitis media. This study used the Strengths and Difficulties Questionnaire (SDQ), completed by both parents and teachers, to assess behavioral domains. Parent-reported outcomes showed increased hyperactivity and emotional difficulties in children who received TTIs, with similar findings observed in both boys and girls. Teacher-reported data echoed these findings but noted worsening specifically among female children. 

Discussion

There was considerable variability in outcome measurement and reporting across the included studies, complicating the interpretation of TTI effects on developmental outcomes. Two randomized controlled trials, Maw et al. [5] and Wilks et al. [7], demonstrated early benefits of TTI. These RCTs represent the highest quality evidence among the studies reviewed. Both used validated developmental assessment tools, the Griffiths Mental Development Scales [5] and the Richman Behavior Checklist [7], which ensured outcomes were evaluated through standardized, clinician-reported measures rather than subjective parental reporting. This methodological rigor improves the reliability and objectivity of their findings. Notably, the age range of participants (2-5 years) corresponds to a sensitive period for developmental progress, making these cohorts especially relevant. Improvements were observed in domains such as personal-social skills and verbal comprehension, particularly at nine months post-intervention. Although longer-term outcomes remain less clear, the internal consistency and design quality of these studies support the validity of their short-term findings. In a field marked by heterogeneity in study design and outcome reporting, these RCTs offer a more controlled and interpretable evaluation of TTI and its early developmental impact.

**Table 4:** Study developmental outcomes. TTI: tympanostomy tube insertion.

Author, year	Study type	N	Intervention	Developmental outcome measurement tool	Results
Barry et al., 2019 [17]	Prospective Cohort Study	96	TTI	ECLIPS	TTI improved developmental outcomes
Rach et al., 1991 [18]	Prospective Cohort Study	52	TTI	Reynell Development Language Scales	No change in developmental outcomes
Niclasen et al., 2016 [13]	Retrospective Cohort study	7599	TTI	SDQ Danish	TTI worsened developmental outcomes
Maw et al., 1999 [5]	Randomized Controlled Trial	182	TTI	McCarthy Scales	TTI improved developmental outcomes
Paradise et al., 2003, 2001, 2007, 2005 [9-12]	Prospective Cohort Study	6350	TTI	McCarthy Scales	No change in developmental outcomes
Rovers et al., 2001 [6]	Randomized Controlled Trial	1174	TTI	Erikson Scales	No change in developmental outcomes
Wilks et al., 2000 [7]	Randomized Controlled Trial	182	TTI	Richman Behavior Checklist	TTI improved developmental outcomes
Schidler et al., 1997 [16]	Prospective Cohort Study	1328	TTI	Language Ability Test	No change in developmental outcomes
Peters et al., 1994 [15]	Prospective Cohort Study	270	TTI	Reading tests/Spelling tests	No change in developmental outcomes
Herzog et al., 2020 [14]	Prospective Cohort Study	52877	TTI	SDQ Danish	No change in developmental outcomes

In contrast, nine prospective cohort studies [9-12,14-18] and one additional RCT [6] found no changes in developmental outcomes following TTI. However, these studies had several limitations that may have influenced these findings. Many employed broader age ranges, often extending beyond the most critical developmental windows, potentially obscuring benefits that might be more detectable in younger children. Others relied on parental or retrospective reporting, which is subject to recall and expectation bias, particularly in the absence of blinded outcome assessment. The developmental tools used in some studies were not tailored to the specific language, behavioral, or cognitive domains most affected by OME, reducing the sensitivity to detect modest but meaningful changes. Additionally, few studies accounted for the duration or severity of effusion prior to intervention, or for socioeconomic and environmental modifiers of developmental outcomes. These methodological shortcomings limit the ability to draw firm conclusions and highlight the need for future high-quality studies focused on well-defined subgroups, timely intervention, and standardized outcome measures.

In contrast, the more recent study by Niclasen et al. [13] reported statistically significant worsening in behavioral outcomes following TTI, particularly among children with four or more episodes of otitis media. Several key limitations reduce the interpretability and generalizability of these results. The study’s reliance on subjective reporting by parents and teachers introduces potential bias, particularly in the absence of standardized observational assessments. Additionally, the wide age range included in the cohort (11 months to 15 years) introduces significant developmental heterogeneity. The comparison groups were restricted to children with ≥4 episodes of OM, limiting applicability to the broader population, especially given the lack of detail about the maximum number or timing of episodes. These methodological limitations, combined with the observational nature of the data, weaken the conclusions regarding harm from TTI.

Our review should be considered in light of several limitations: (1) demographic, socioeconomic status (SES), and comorbid conditions are not frequently reported; (2) notable variation in the assessed length of the developmental period post-intervention (ranging from 3 months to 11 years); (3) DMIs rarely overlap and vary in both reliability and validity; and (4) a paucity of rigorous studies. Of these, the most pronounced short coming was inconsistent reporting of patient demographics, SES, and comorbid conditions. Studies infrequently reported SES and demographic characteristics (Table 3). SES factors, such as family income, parental education, and access to healthcare, can significantly influence a child's development, including language acquisition, cognitive abilities, and emotional well-being. SES can directly affect a child's access to early interventions, quality of care, and developmental support, which in turn could skew results if not adequately adjusted for in the analysis [19]. Without consistent data on SES, it is challenging to draw clear conclusions regarding the efficacy of TTI on child development across different socioeconomic groups. In the same light, infrequent reporting of comorbid conditions such as obstructive sleep apnea (OSA) impacted our analyses [19]. Only one study [7] addressed comorbid conditions, despite OSA also having shown to have significant influences on developmental outcomes in the pediatric populations, such as increased inattention and hyperactivity [20]. OME is often diagnosed along with OSA, and children with both conditions may experience more severe developmental challenges, including speech and language delays, behavioral issues, and difficulties with attention and learning [1]. Failure to account for these comorbidities further introduces inconsistency into the findings, as children with additional health challenges may respond differently to treatments like TTI. Furthermore, this review only analyzed studies performing TTI with no other interventions. As a result, patients who underwent TTI with adenoidectomy or adenotonsillectomy may have different trends in developmental outcomes. 

## Conclusions

Developmental outcomes are complex and multifaceted, encompassing cognitive, emotional, and social domains. Our analysis revealed mixed results: RCTs showed improvement, while cohort studies showed no significant difference. The lack of consistency in patient demographics, outcome measurement tools, and the reporting of comorbidities complicates drawing definitive conclusions about the impact of TTI on developmental outcomes. TTI aims to alleviate hearing loss and reduce the frequency of middle ear infections, both of which can impact language, cognitive function, and behavioral health. However, its effects on development vary across children and can be influenced by factors such as the timing of placement, the severity and duration of OME, the child’s overall health, socioeconomic status, and environmental factors. More standardized research is needed to elucidate the role of TTI on developmental outcomes, including patient demographic, SES, and comorbid factors. Future studies investigating the developmental impact of TTI in pediatric OM should consider using a validated developmental tool. This tool should objectively assess clearly defined domains, such as speech and language acquisition, social behavior, and cognitive and motor development.
